# Aberrations in stimulated emission depletion (STED) microscopy

**DOI:** 10.1016/j.optcom.2017.06.037

**Published:** 2017-12-01

**Authors:** Jacopo Antonello, Daniel Burke, Martin J. Booth

**Affiliations:** a Centre for Neural Circuits and Behaviour, University of Oxford, Mansfield Road, Oxford, OX1 3SR, UK; b Department of Engineering Science, University of Oxford, Parks Road, Oxford, OX1 3PJ, UK

**Keywords:** 00-01, 99-00, Aberration correction, Adaptive optics, High numerical aperture optics, Stimulated emission depletion microscopy, Super-resolution microscopy

## Abstract

Like all methods of super-resolution microscopy, stimulated emission depletion (STED) microscopy can suffer from the effects of aberrations. The most important aspect of a STED microscope is that the depletion focus maintains a minimum, ideally zero, intensity point that is surrounded by a region of higher intensity. It follows that aberrations that cause a non-zero value of this minimum intensity are the most detrimental, as they inhibit fluorescence emission even at the centre of the depletion focus. We present analysis that elucidates the nature of these effects in terms of the different polarisation components at the focus for two-dimensional and three-dimensional STED resolution enhancement. It is found that only certain low-order aberration modes can affect the minimum intensity at the Gaussian focus. This has important consequences for the design of adaptive optics aberration correction systems.

## Highlights

• Aberrations disrupt the performance of stimulated emission depletion microscopy.
• 2D and 3D STED are most affected by different sets of aberrations.
• Some low-order aberrations induce axial and lateral shifts.

## 1 Introduction

In stimulated emission depletion (STED) microscopy, super-resolution is achieved by confining fluorescence emission to a region much smaller than the focussed laser beam that is used to excite the fluorophores [1], [2]. This super-resolution effect is achieved through the use of a depletion focus, which is designed to have a minimum – ideally zero – intensity point that is surrounded by high intensity light. The depletion focus suppresses fluorescence at the periphery of the excitation focus by forcing excited fluorophores back to the ground state via stimulated emission. The contrast of this depletion beam – especially through the value of the intensity minimum – is critical in maintaining the super-resolution properties of the microscope [3]. Whilst aberrations affect in general all beams paths (excitation, emission, and depletion) of the STED microscope, for small aberration amplitudes the effects on performance are dominated by the distortion of the depletion beam [4], [5], [6], [7], [8].

Understanding the effects of aberrations on the image formation process is important in informing the design of adaptive optics systems that are effective in suppressing system and specimen induced aberrations [9], [10], [11], [12], [13]. The effective point-spread function (PSF) of the STED microscope can be expressed as

(1) $$h_{\text{STED}}\left(x\right)=\eta \left(x\right)h_{\text{conf}}\left(x\right),$$

where x is the position vector, $h_{\text{conf}}$ is the PSF of the underlying confocal microscope, and η is the suppression factor [14], given by

(2) $$\eta \left(x\right)=\exp\left(\frac{-\ln\left(2\right)I_{\text{dep}}\left(x\right)}{I_{s}}\right),$$

where $I_{\text{dep}}$ is the intensity of the depletion focus and $I_{s}$ is the intensity value at which fluorescence emission drops to one half of its maximum value. In a neighbourhood of the Gaussian focus, the intensity profile of the depletion beam can be approximated by a quadratic function $I_{\text{dep}}\left(x\right)\approx I_{0}\left(a+bx^{2}\right)$, where for simplicity we consider only the variation in the x direction. The values of the parameters $I_{0}$, a, and b will depend upon the aberrations present in the system. By replacing this expression in Eq. (2), the suppression factor then becomes

(3) $$\eta \left(x\right)=\exp\left(-ac\right)\exp\left(-bcx^{2}\right),$$

where $c=\ln\left(2\right)I_{0}∕I_{s}$. For high resolution enhancement, the depletion intensity would be set so that c≫1. From Eq. (3) it is clear that a deviation of a from zero could significantly reduce the fluorescence emission measured by the STED microscope, by inhibiting emission at the Gaussian focus. Such an undesirable depletion effect at the centre can be more disruptive than a reduction in the efficiency of the depletion in the surrounding area – modelled by variations in b – which would only affect the resolution achieved by the STED microscope. It is therefore important to investigate the conditions under which the intensity at the Gaussian focus deviates from zero.

Deng et al. [6], [7] studied the effects of aberrations on 2D and 3D STED microscopes by considering the Seidel coefficients for spherical, coma, and astigmatism aberrations. Through numerical evaluations of the corresponding diffraction integrals, they observed various distortions of the focus caused by these aberrations. In this paper we complement their numerical findings by deriving mathematical expressions that model the effects of aberrations on the zero of the depletion focus. This analysis is facilitated by representing the electric field at the focus in terms of its circular polarisation components. It is found that the zero of the depletion focus is affected in different ways by different aberration modes, according to their degree of azimuthal symmetry.

## 2 Focussing model

### 2.1 Representation of the focal field

The intensity of the depletion focus is proportional to the modulus squared of the electric field vector $E=\left(E_{x},E_{y},E_{z}\right)$, defined with respect to the Cartesian coordinates (x,y,z). Our subsequent analysis is simplified however if we redefine the field vector in terms of circular polarisation components: $E_{l}=\left(E_{x}-iE_{y}\right)∕\sqrt{2}$ and $E_{r}=\left(E_{x}+iE_{y}\right)∕\sqrt{2}$, where the subscripts l and r refer, respectively, to the left and right circularly polarised components. As both the Cartesian and circular polarisation components form orthogonal bases, we have that the intensity of the depletion focus is proportional to I, where

(4) $$I={\left|E\right|}^{2}={\left|E_{x}\right|}^{2}+{\left|E_{y}\right|}^{2}+{\left|E_{z}\right|}^{2}={\left|E_{l}\right|}^{2}+{\left|E_{r}\right|}^{2}+{\left|E_{z}\right|}^{2}.$$

The most common configurations of the STED microscope require circularly polarised illumination in the pupil [15]. The corresponding electric field at the focus can be computed as [16], [17], [18] :

(5) $$\left[\begin{array}{c} E_{x}\\ E_{y}\\ E_{z} \end{array}\right]=\int_{\theta =0}^{\alpha }\int_{\phi =0}^{2\pi }A\left(\theta \right)\sqrt{\cos\theta }\left[\begin{array}{c} \cos\theta +1+\left(\cos\theta -1\right)e^{i2\phi }\\ i\left(\cos\theta +1\right)-i\left(\cos\theta -1\right)e^{i2\phi }\\ -2\sin\theta e^{i\phi } \end{array}\right]\;\times T\left(\theta ,\phi \right)P\left(\theta ,\phi \right)\exp\left[ik\left(\rho \sin\theta \cos\left(\phi -\xi \right)+z\cos\theta \right)\right]\sin\theta \;d\phi \;d\theta ,$$

where k=n2π∕λ, n is the index of refraction, and λ is the wavelength. A(θ) is the illumination profile from the laser, which is assumed to vary along the polar coordinate θ only. T(θ,ϕ) is the phase mask for generation of the STED depletion focus and P(θ,ϕ) is the generalised pupil function (GPF) [19], which accounts for the effects of aberrations. The $\sqrt{\cos\theta }$ is the apodisation term for an objective lens obeying the sine condition [16]. The integration takes place over a spherical cap described by the coordinates (θ,ϕ), where the upper limit is α=arcsin(NA∕n) and NA is the numerical aperture of the objective. Note that we have omitted from Eq. (5) some constant factors that will not affect our subsequent analysis.

Eq. (5) can be expressed in a more convenient form by outlining the circular polarisation components of the electric field vector at the focus:

(6) $$U\cdot E=\left[\begin{array}{c} E_{l}\\ E_{r}\\ E_{z} \end{array}\right]=\int_{\theta =0}^{\alpha }\int_{\phi =0}^{2\pi }A\left(\theta \right)\sqrt{\cos\theta }\left[\begin{array}{c} \sqrt{2}\left(\cos\theta +1\right)\\ \sqrt{2}\left(\cos\theta -1\right)e^{i2\phi }\\ -2\sin\theta e^{i\phi } \end{array}\right]\;\times T\left(\theta ,\phi \right)P\left(\theta ,\phi \right)\exp\left[ik\left(\rho \sin\theta \cos\left(\phi -\xi \right)+z\cos\theta \right)\right]\sin\theta \;d\phi \;d\theta ,$$

where U is an appropriate unitary matrix corresponding to the transformation described at the beginning of this section. In the following sections, we study the behaviour of Eq. (6) for aberrations that have different azimuthal orders. Many properties can be determined by examining the behaviour of the integral in Eq. (6) with respect to the azimuthal coordinate ϕ. In particular, we shall see that the response of the STED microscope to different aberrations can be categorised according to the azimuthal variation of the aberration mode.

### 2.2 Aberration expansion

We assume that the GPF represents purely a phase aberration and that amplitude variations can be neglected. Accordingly, one has $P\left(\theta ,\phi \right)=\exp\left[i\Phi \left(\theta ,\phi \right)\right]$, where Φ(θ,ϕ) is the phase aberration function, which is expanded as a series of different azimuthal orders:

(7) $$\Phi \left(\theta ,\phi \right)=f_{0}\left(\theta \right)+\overset{\infty }{\underset{m=1}{\sum }}\left(f_{m}\left(\theta \right)\cos\left(m\phi \right)+g_{m}\left(\theta \right)\sin\left(m\phi \right)\right),$$

where $f_{0}$, $f_{m}$ and $g_{m}$ are arbitrary functions. One could further expand such functions using the normalised radial coordinate in the pupil r=sin(θ)∕sin(α) to recover the conventional decomposition of the phase into Zernike polynomials [20]. For the present analysis, however, it is more convenient to employ the expansion of Eq. (7). The first term $f_{0}\left(\theta \right)$ represents the aberration due to Zernike polynomials of azimuthal order zero, which includes defocus, spherical aberration, and higher order spherical aberrations. The other aberration modes are grouped according to their azimuthal orders, e.g., coma, tip, and tilt for m=1, and astigmatism for m=2.

For small aberrations, the GPF can be approximated by the first two terms of a Taylor expansion, i.e., 

(8) $$P\left(\theta ,\phi \right)\approx 1+i\left[f_{0}\left(\theta \right)+\overset{\infty }{\underset{m=1}{\sum }}\left(f_{m}\left(\theta \right)\cos\left(m\phi \right)+g_{m}\left(\theta \right)\sin\left(m\phi \right)\right)\right],$$

which corresponds to an expansion into complex-valued Zernike polynomials [21], [22].

### 2.3 STED phase masks

**Fig. 1:**
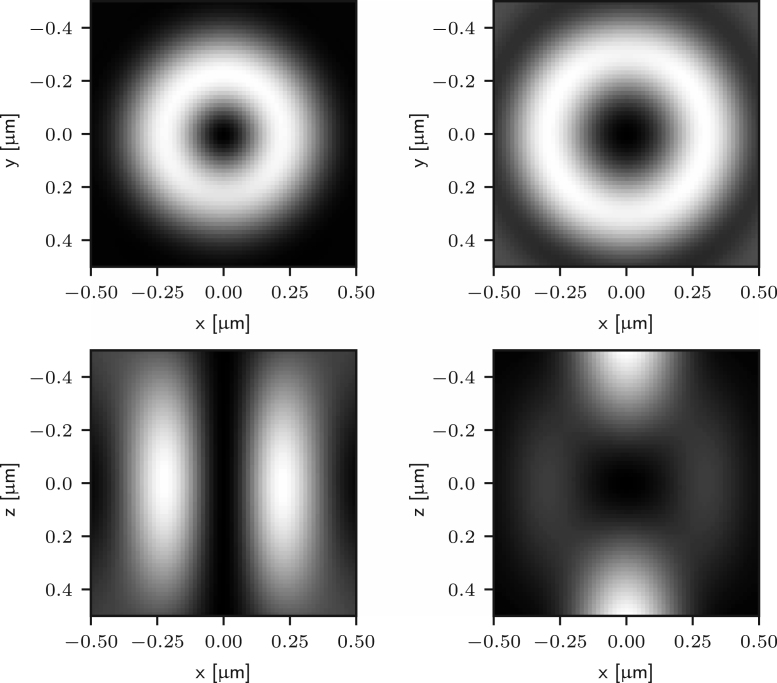
Sections of the depletion point-spread functions along the xy plane (top row) and xz plane (bottom row) for 2D STED (left column) and 3D STED (right column). Each image uses an independent colour map to enhance the contrast. The sections were obtained by numerically integrating Eq. (5) where n=1.518, NA=1.4, and λ=775nm.

The 2D and 3D STED configurations require different phase masks T(θ,ϕ) to create the depletion foci. For 2D STED we consider the helicoidal phase pattern described by

(9) $$T_{2}\left(\phi \right)=e^{i\phi },$$

which results in the depletion pattern depicted on the left column in Fig. 1. For 3D STED, we use the pi-step phase mask defined by

(10) $$T_{3}\left(\theta \right)=\left\{\begin{array}{cc} -1 & \theta \leq \beta \\ 1 & \theta >\beta , \end{array}\right)$$

where β is chosen to ensure destructive interference at the centre of the focus. In practice, β depends upon the illumination profile A(θ) and is often tuned empirically. The depletion focus obtained with $T_{3}$ is shown on the right column of Fig. 1.

## 3 Focal distribution in aberration-free 2D STED microscopes

For the 2D STED microscope we use the phase mask $T_{2}$ defined in Eq. (9). The focal field without aberrations is found by setting P(θ,ϕ)=1:

(11) $$\left[\begin{array}{c} E_{l}\\ E_{r}\\ E_{z} \end{array}\right]=\int_{\theta =0}^{\alpha }\int_{\phi =0}^{2\pi }A\left(\theta \right)\sqrt{\cos\theta }\left[\begin{array}{c} \sqrt{2}\left(\cos\theta +1\right)e^{i\phi }\\ \sqrt{2}\left(\cos\theta -1\right)e^{i3\phi }\\ -2\sin\theta e^{i2\phi } \end{array}\right]\;\times \exp\left[ik\left(\rho \sin\theta \cos\left(\phi -\xi \right)+z\cos\theta \right)\right]\sin\theta \;d\phi \;d\theta .$$

One can compute the inner integrals in dϕ directly in Eq. (11), using the identity $\int_{0}^{2\pi }\exp\left[iw\cos\left(\phi -\xi \right)+im\phi \right]\;d\phi =i^{m}2\pi e^{im\xi }J_{m}\left(w\right)$ from [16], where $J_{m}$ is the mth Bessel function of the first kind. In doing so, one finds

(12) $$\int_{0}^{2\pi }\left[\begin{array}{c} e^{i\phi }\\ e^{i3\phi }\\ e^{i2\phi } \end{array}\right]\exp\left[ik\rho \sin\theta \cos\left(\phi -\xi \right)\right]\;d\phi =2\pi \left[\begin{array}{c} ie^{i\xi }J_{1}\left(k\rho \sin\theta \right)\\ -ie^{i3\xi }J_{3}\left(k\rho \sin\theta \right)\\ -e^{i2\xi }J_{2}\left(k\rho \sin\theta \right) \end{array}\right].$$

Since the Bessel functions on the right-hand side of Eq. (12) evaluate to zero for ρ=0, one can deduce that the intensity of the depletion focus vanishes at the Gaussian focus, as required for the super-resolution effect. Replacing Eq. (12) into Eq. (11) allows us to write

(13) $$\left[\begin{array}{c} E_{l}\\ E_{r}\\ E_{z} \end{array}\right]=2\pi \int_{0}^{\alpha }A\left(\theta \right)\sqrt{\cos\theta }\left[\begin{array}{c} i\sqrt{2}e^{i\xi }\left(\cos\theta +1\right)J_{1}\left(k\rho \sin\theta \right)\\ -i\sqrt{2}e^{i3\xi }\left(\cos\theta -1\right)J_{3}\left(k\rho \sin\theta \right)\\ 2e^{i2\xi }\sin\theta J_{2}\left(k\rho \sin\theta \right) \end{array}\right]e^{ikz\cos\theta }\sin\theta \;d\theta .$$

In a neighbourhood of ρ=0, one can use the small argument approximation [23] $J_{m}\left(w\right)\approx w^{m}∕\left(2^{m}m!\right)$. Once the integrals in dθ are computed, one can see that the behaviour of the components of the electric field is approximated by

(14) $$\left|E_{l}\right|\propto \rho \;\left|E_{r}\right|\propto \rho^{3}\;\left|E_{z}\right|\propto \rho^{2}.$$

From Eq. (4) we find that, at first order approximation, $I\propto \rho^{2}$ due variations in $E_{l}$. It follows that the lateral resolution achieved by the STED microscope is predominantly affected by variations of the left circularly polarised component of the electric field at the focus.

## 4 Effects of aberrations on the intensity zero in 2D STED microscopes

We study the effects that aberrations have on the 2D depletion zero by considering the variations of the electric field along the optical axis, i.e., for ρ=0. Setting $T=T_{2}$ in Eq. (6) we obtain

(15) $$\left[\begin{array}{c} E_{l}\\ E_{r}\\ E_{z} \end{array}\right]=\int_{\theta =0}^{\alpha }\int_{\phi =0}^{2\pi }P\left(\theta ,\phi \right)\left[\begin{array}{c} \sqrt{2}\left(\cos\theta +1\right)e^{i\phi }\\ \sqrt{2}\left(\cos\theta -1\right)e^{i3\phi }\\ -2\sin\theta e^{i2\phi } \end{array}\right]q_{0}\left(\theta ,z\right)\;d\phi \;d\theta ,$$

where $q_{0}\left(\theta ,z\right)=A\left(\theta \right)\sqrt{\cos\theta }\exp\left(ikz\cos\theta \right)\sin\theta$.

We first consider the aberrations of azimuthal order zero in Eq. (7), which are due to $f_{0}\left(\theta \right)$ only. In this case, P(θ,ϕ) in Eq. (15) is replaced by $P\left(\theta \right)=\exp\left[if_{0}\left(\theta \right)\right]$. Such aberrations correspond to arbitrary combinations of Zernike defocus and spherical aberration terms. Since P(θ) does not depend on ϕ, Eq. (12) still applies and we can conclude that, for ρ=0, such aberrations do not affect the zero of the electric field along the optical axis. This implies that the performance of 2D STED microscopes is robust with respect to aberrations of azimuthal order zero — although such aberrations affect the depletion efficiency of the ring surrounding the zero, they do not introduce any unwanted depletion at the Gaussian focus itself. This observation is confirmed numerically by computing Eq. (5) using different amounts of Zernike spherical aberration, as reported in Fig. 2 (a), where it can be seen that the zero is maintained.

Instead, when considering the non-zero azimuthal order terms $f_{m}\left(\theta \right)\cos\left(m\phi \right)$ and $g_{m}\left(\theta \right)\sin\left(m\phi \right)$ in Eq. (7), we restrict ourselves to aberrations of small amplitude, so that we can use the approximation of the GPF found in Eq. (8). The constant term 1 and the term $if_{0}\left(\theta \right)$ in Eq. (8) correspond, respectively, to the cases that have already been addressed in the previous section and in the previous paragraph. At present, we consider the case $P\left(\theta ,\phi \right)\approx if_{m}\left(\theta \right)\cos\left(m\phi \right)$ and replace it into Eq. (15), which leads to

(16) $$\left[\begin{array}{c} E_{l}\\ E_{r}\\ E_{z} \end{array}\right]=\left\{\int_{0}^{\alpha }\left[\begin{array}{c} \sqrt{2}\left(\cos\theta +1\right)\\ \sqrt{2}\left(\cos\theta -1\right)\\ -2\sin\theta \end{array}\right]if_{m}\left(\theta \right)q_{0}\left(\theta ,z\right)\;d\theta \right\}⊙\left\{\int_{0}^{2\pi }\left[\begin{array}{c} e^{i\phi }\\ e^{i3\phi }\\ e^{i2\phi } \end{array}\right]\cos\left(m\phi \right)\;d\phi \right\},$$

where ⊙ represents the Hadamard (element wise) product between the two vectors.

It is apparent that the integrals in ϕ may now lead to non-zero values of the field components for the following situations: for m=1 (coma), $E_{l}$ may be non-zero; for m=2 (astigmatism), $E_{z}$ may be non-zero; for m=3 (trefoil), $E_{r}$ may be non-zero. In all such cases m=1,2,3 the on-axis intensity can be non-zero. On the other hand, for all other azimuthal orders m≥4 the on-axis intensity remains zero. Similar results would also be obtained using aberration modes of the form $g_{m}\left(\theta \right)\sin\left(m\phi \right)$. This observation can also be made by looking at Fig. 2 (b)–(d), where one can see that small amounts of Zernike coma, astigmatism, and trefoil lead to non-zero intensity at the Gaussian focus. For the case of m=1 (coma) the on-axis intensity is non-zero. Nevertheless it has already been shown that [24], at first order approximation, the zero is still present but has been shifted laterally. This can be seen in Fig. 2 (b), where the minimum of the intensity is moved off axis by small amounts of Zernike coma.

To further validate the analysis reported in this section we numerically evaluate the optical transfer function (OTF) of the 2D STED microscope when different aberrations are present. The OTF, which can be computed from the equations outlined in [14], [25], takes into account the combined effect of the aberrations on the excitation, depletion, and detection beams. The results are reported in Fig. 3, where we have assumed an excitation wavelength of 650 nm, a detection wavelength of 685 nm, and aberrations with an rms of 61.67 nm, which corresponds to 0.5 rad for the depletion wavelength. As a result of the aberrations, the OTF shows a reduction in the gain especially for high spatial frequencies. It can be seen that the detrimental effect of spherical ($Z_{4}^{0}$) and coma ($Z_{3}^{1}$) on the OTF is less prominent than that of astigmatism ($Z_{2}^{2}$), since this latter causes the zero to fill in at first order, contrary to the case for spherical and coma. Furthermore, it is worth noting that although trefoil ($Z_{3}^{3}$) indeed causes the zero to fill in as discussed above, the effect in this case is much less severe than for astigmatism. This is motivated by the fact that trefoil causes the right circularly polarised component $E_{r}$ to become non-zero at the Gaussian focus. Nevertheless, the magnitude of $E_{r}$ is still much smaller than the magnitude of $E_{l}$ and $E_{z}$, which are dominating due to fact that the illumination in the pupil is left circularly polarised and that a high NA objective is used. Finally, it can be seen that for aberrations where m≥4, such as $Z_{4}^{4}$ in Fig. 3, the detrimental effect is also less severe, as these aberrations do not cause the zero to fill in, which is in accordance to the analysis above.Fig. 2Intensity profile along the x-axis of the 2D STED depletion focus obtained by numerically solving Eq. (5) when different amounts of spherical (a), coma (b), astigmatism (c), and trefoil (d) Zernike aberrations are present. In each case, the thick curves correspond to the aberration-free case. The black and dark grey curves correspond to negative and positive amounts of aberration. All curves are normalised to the maximum value reported for the aberration-free case. The phase and PSF in the xy plane are reported below the intensity profiles, along with the magnitude of the aberration in rad. Note that the extent of the PSFs is the same as that in Fig. 1, and is larger than the extent used in the profile graphs. (a) spherical (m=0) does not affect the on-axis intensity. (b)–(d) coma, astigmatism, and trefoil affect the on-axis intensity. (b) coma shifts the zero at first order approximation.

**Fig. 2(a):**
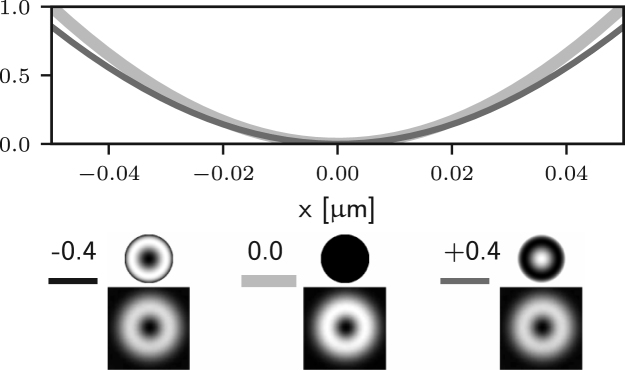
(a) $Z_{4}^{0}$.

**Fig. 2(b):**
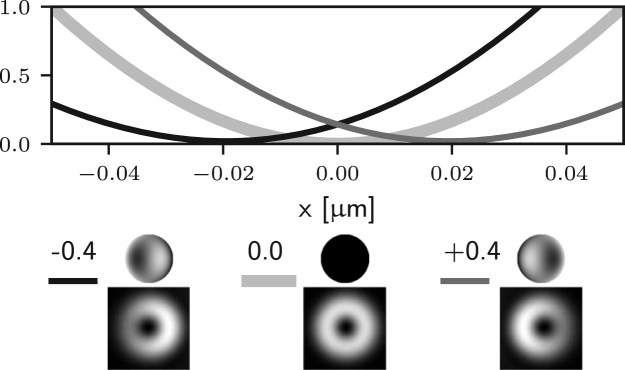
(b) $Z_{3}^{1}$.

**Fig. 2(c):**
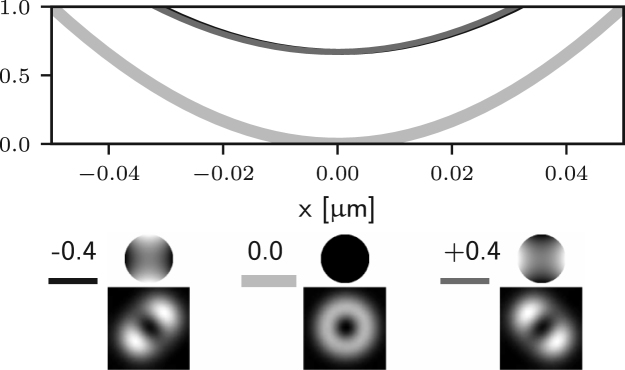
(c) $Z_{2}^{2}$.

**Fig. 2(d):**
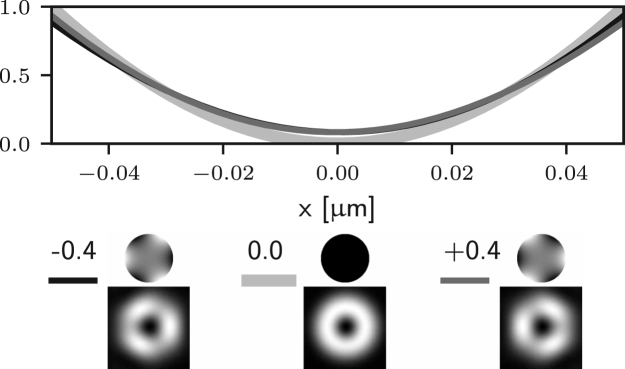
(d) $Z_{3}^{3}$.

**Fig. 3:**
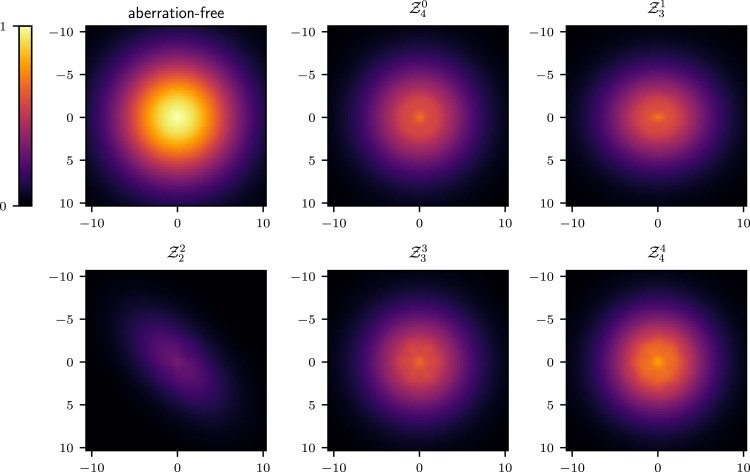
Effects of aberrations on the optical transfer function (OTF) computed for the 2D STED microscope. $Z_{4}^{0}$ spherical; $Z_{3}^{1}$ coma; $Z_{2}^{2}$ astigmatism; $Z_{3}^{3}$ trefoil; $Z_{4}^{4}$ quadrafoil; Units are in $\mu {\text{m}}^{-1}$ and each plot uses the same colour map determined by the aberration-free OTF. At first order approximation, the depletion zero fills in for $Z_{2}^{2}$ and $Z_{3}^{3}$. A severe reduction in the magnitude of the OTF is seen for astigmatism. A less severe effect is found for trefoil, since the corresponding non-zero component of the electric field $E_{r}$ is negligible at the Gaussian focus, see Section 4. At first order approximation, aberrations with m≥4 do not cause the depletion zero to fill in and thus have a less severe effect on the OTF.

## 5 Focal distribution in aberration-free 3D STED microscopes

For the 3D STED microscope, we use the phase mask defined in Eq. (10). The focal field without aberrations is found as:

(17) $$\left[\begin{array}{c} E_{l}\\ E_{r}\\ E_{z} \end{array}\right]=\int_{\theta =0}^{\alpha }\int_{\phi =0}^{2\pi }A\left(\theta \right)\sqrt{\cos\theta }\left[\begin{array}{c} \sqrt{2}\left(\cos\theta +1\right)\\ \sqrt{2}\left(\cos\theta -1\right)e^{i2\phi }\\ -2\sin\theta e^{i\phi } \end{array}\right]\;\times T_{3}\left(\theta \right)\exp\left[ik\left(\rho \sin\theta \cos\left(\phi -\xi \right)+z\cos\theta \right)\right]\sin\theta \;d\phi \;d\theta .$$

As performed in the previous sections, we can solve the inner integrals in dϕ, which leads to

(18) $$\left[\begin{array}{c} E_{l}\\ E_{r}\\ E_{z} \end{array}\right]=2\pi \int_{0}^{\alpha }A\left(\theta \right)\sqrt{\cos\theta }\left[\begin{array}{c} \sqrt{2}\left(\cos\theta +1\right)J_{0}\left(k\rho \sin\theta \right)\\ -\sqrt{2}e^{i2\xi }\left(\cos\theta -1\right)J_{2}\left(k\rho \sin\theta \right)\\ -i2e^{i\xi }\sin\theta J_{1}\left(k\rho \sin\theta \right) \end{array}\right]T_{3}\left(\theta \right)e^{ikz\cos\theta }\sin\theta \;d\theta .$$

When considering the Gaussian focus (ρ=0 and z=0), one can see that the components $E_{r}$ and $E_{z}$ of Eq. (18) are guaranteed to vanish due to the corresponding Bessel functions $J_{2}$ and $J_{1}$. Instead, to make the $E_{l}$ component vanish, the following condition must be satisfied:

(19) $$\int_{0}^{\alpha }\;A\left(\theta \right)\sqrt{\cos\theta }\left(\cos\theta +1\right)T_{3}\left(\theta \right)\sin\theta \;d\theta =0.$$

When A(θ) has a simple form, for example it is a constant function, Eq. (19) can be solved as a function of cosβ, where β is the parameter of the pi-step phase mask in Eq. (10). It can be seen that, contrary to the case for 2D STED, the existence of the zero in 3D STED is also conditional to an equation that involves θ and the radial apodisation function A(θ). This explains why, experimentally, 3D STED has been found to be more susceptible to aberrations than 2D STED [10].

Using the small argument approximation for the Bessel function, we find that in the plane perpendicular to the optical axis the polarisation components vary as

(20) $$\left|E_{l}\right|\propto \rho^{2}\;\left|E_{r}\right|\propto \rho^{2}\;\left|E_{z}\right|\propto \rho ,$$

where the variation of $E_{l}$ with ρ was determined by the second term in the Taylor series of $J_{0}$. Using Eq. (4) we find that $I\propto \rho^{2}$ due the variation in $E_{z}$. It follows that the lateral resolution is determined by the axial polarisation component, which is non-negligible when using high NA objectives.

We further investigate the enhancement of resolution along the optical axis by determining the variation of E as a function of z. For ρ=0, both $E_{r}$ and $E_{z}$ are zero in Eq. (18), and we only need to concern ourselves with the integral in dθ of $E_{l}$. For small z, we can approximate the Jacobi–Anger identity [23] $\exp\left(ikz\cos\theta \right)=\sum_{m=-\infty }^{\infty }i^{m}J_{m}\left(kz\right)\exp\left(im\theta \right)$ with its leading terms found for m=0 and m=±1, so that $E_{l}$ is approximately proportional to

(21) $$\int_{0}^{\alpha }\;A\left(\theta \right)\sqrt{\cos\theta }\left(\cos\theta +1\right)T_{3}\left(\theta \right)\left[J_{0}\left(kz\right)+i2J_{1}\left(kz\right)\cos\theta \right]\sin\theta \;d\theta .$$

By considering the small argument approximations of the Bessel functions in Eq. (21) and the condition in Eq. (19), we have that

(22) $$\left|E_{l}\right|\propto z\;\left|E_{r}\right|=0\;\left|E_{z}\right|=0.$$

It follows that the axial resolution is determined by the $E_{l}$ component and $I\propto z^{2}$, where we remark that $E_{r}=E_{z}=0$ also holds for all values of z, not only small values.

## 6 Effects of aberrations on the intensity zero in 3D STED microscopes

Using the phase mask $T_{3}$ defined in Eq. (10), the electric field at ρ=0 in the presence of aberrations is given by

(23) $$\left[\begin{array}{c} E_{l}\\ E_{r}\\ E_{z} \end{array}\right]=\int_{\theta =0}^{\alpha }\int_{\phi =0}^{2\pi }A\left(\theta \right)\sqrt{\cos\theta }\left[\begin{array}{c} \sqrt{2}\left(\cos\theta +1\right)\\ \sqrt{2}\left(\cos\theta -1\right)e^{i2\phi }\\ -2\sin\theta e^{i\phi } \end{array}\right]\;\times T_{3}\left(\theta \right)P\left(\theta ,\phi \right)e^{iz\cos\theta }\sin\theta \;d\phi \;d\theta .$$

We consider again the effects of each term in Eq. (7) individually, starting from $f_{0}\left(\theta \right)$. Since $f_{0}\left(\theta \right)$ does not depend on ϕ we can conclude that both $E_{r}$ and $E_{z}$ vanish, based on the discussion in the previous section. However the component $E_{l}$ may only vanish provided

(24) $$\int_{0}^{\alpha }\;A\left(\theta \right)\sqrt{\cos\theta }\left(\cos\theta +1\right)T_{3}\left(\theta \right)\exp\left[i\left(f_{0}\left(\theta \right)+kz\cos\theta \right)\right]\sin\theta \;d\theta =0.$$

This condition is not guaranteed to hold for z=0 or in general elsewhere along the optical axis. Therefore aberrations with azimuthal order zero may indeed affect the zero of 3D STED microscopes. One notable example consists of aberration-free defocussing for a high NA objective. In this case the corresponding phase is given by $f_{0}\left(\theta \right)=d\cos\theta$, where d is the magnitude of the defocus. As shown in [26], one can decompose this phase term into a combination of Zernike piston, defocus, and spherical aberrations of all orders. This implies that the axial position of the zero will be affected by small amounts of spherical aberration, as shown in Fig. 4 (a).

We now consider the next group of aberrations in Eq. (7), which have non-zero azimuthal order and concern term $f_{m}\left(\theta \right)\cos\left(m\phi \right)$. For simplicity, we restrict ourselves to small amplitude aberrations and apply the approximation in Eq. (8) for the GPF. Again, we do not consider the constant term 1 and term if(θ), which have been discussed in the previous section and, in part, within the previous paragraph. Replacing P(θ,ϕ) with $if_{m}\left(\theta \right)\cos\left(m\phi \right)$ in Eq. (23) leads to

(25) $$\left[\begin{array}{c} E_{l}\\ E_{r}\\ E_{z} \end{array}\right]=\left\{\int_{0}^{\alpha }\left[\begin{array}{c} \sqrt{2}\left(\cos\theta +1\right)\\ \sqrt{2}\left(\cos\theta -1\right)\\ -2\sin\theta \end{array}\right]f_{m}\left(\theta \right)q_{2}\left(\theta \right)\;d\theta \right\}⊙\left\{\int_{0}^{2\pi }\left[\begin{array}{c} 1\\ e^{i2\phi }\\ e^{i\phi } \end{array}\right]\cos\left(m\phi \right)\;d\phi \right\},$$

where $q_{2}\left(\theta \right)=iA\left(\theta \right)\sqrt{\cos\theta }T_{3}\left(\theta \right)\sin\theta$.

It is apparent that two of the integrals in ϕ may now lead to non-zero values of the field components in the following situations: for m=1 (coma), $E_{z}$ may be non-zero; for m=2 (astigmatism), $E_{r}$ may be non-zero. Examples of the effects of such aberrations are given in Figs. 5 (b)–(c) and 4 (b)–(c), where it is shown that the on-axis intensity becomes non-zero for such aberrations. For the specific case m=1, it has already been shown that a small amplitude of a coma-like mode does not actually destroy the zero, but simply shifts it off axis [24], as shown in Fig. 5 (b). Taking into account the zero azimuthal order case discussed earlier, one can conclude that for m=0,1,2 the intensity at the Gaussian focus can be non-zero. For small amplitudes of all other aberration modes (m≥3) the on-axis intensity remains approximately zero, as shown in Figs. 5 (d) and 4 (d). Similar results would also be obtained using aberration modes of the form $g_{m}\left(\theta \right)\sin\left(m\phi \right)$.

Fig. 4Intensity profile along the z-axis of the 3D STED depletion focus obtained by numerically solving Eq. (5) when different amounts of spherical (a), coma (b), astigmatism (c), and trefoil (d) Zernike aberrations are present. See the caption of Fig. 2 for the legend. Note that the extent of the PSFs is the same as that in Fig. 1. (a) spherical aberration (m=0) approximately shifts the zero along the optical axis. (b) and (c) coma and astigmatism (m=1 and m=2) fill in the on-axis intensity. (d) trefoil (m=3) does not affect the zero at first order approximation.

**Fig. 4(a):**
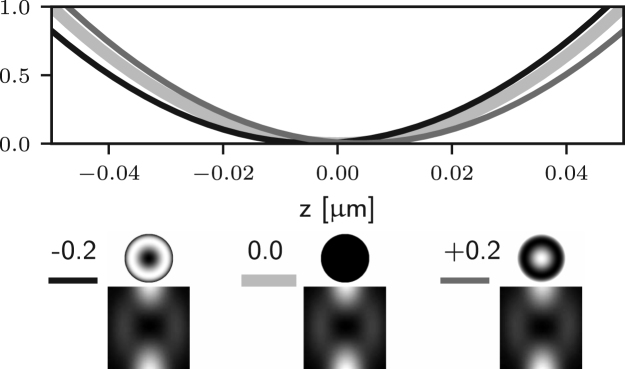
(a) $Z_{4}^{0}$.

**Fig. 4(b):**
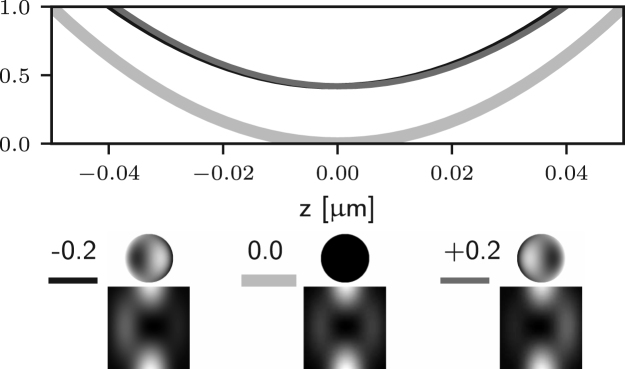
(b) $Z_{3}^{1}$.

**Fig. 4(c):**
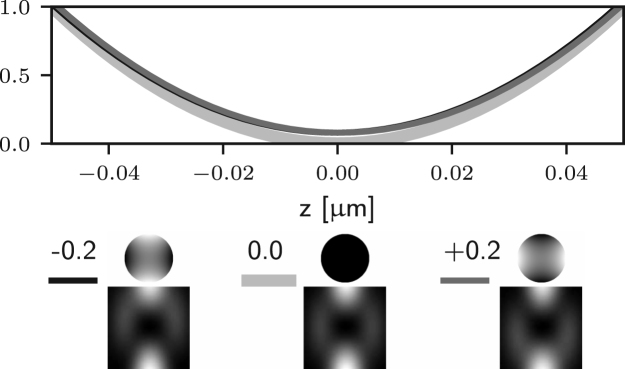
(c) $Z_{2}^{2}$.

**Fig. 4(d):**
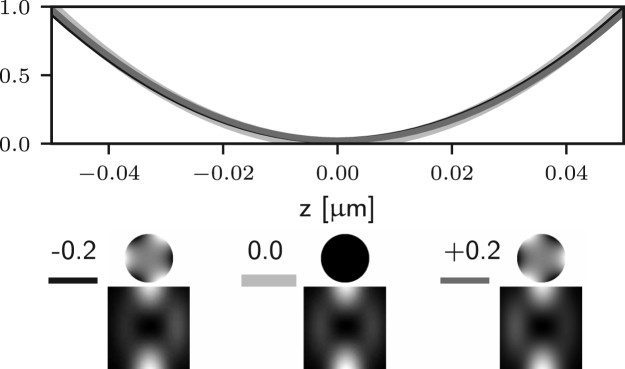
(d) $Z_{3}^{3}$.Fig. 5Intensity profile along the x-axis of the 3D STED depletion focus obtained by numerically solving Eq. (5) when different amounts of spherical (a), coma (b), astigmatism (c), and trefoil (d) Zernike aberrations are present. See the caption of Fig. 2 for the legend. Note that the extent of the PSFs is the same as that in Fig. 1 (a) spherical aberration (m=0) approximately shifts the zero along the optical axis, see Fig. 4. (b) and (c) coma and astigmatism (m=1 and m=2) fill in the on-axis intensity. (b) coma approximately shifts the zero along x. (d) trefoil (m=3) does not affect the zero at first order approximation.

**Fig. 5(a):**
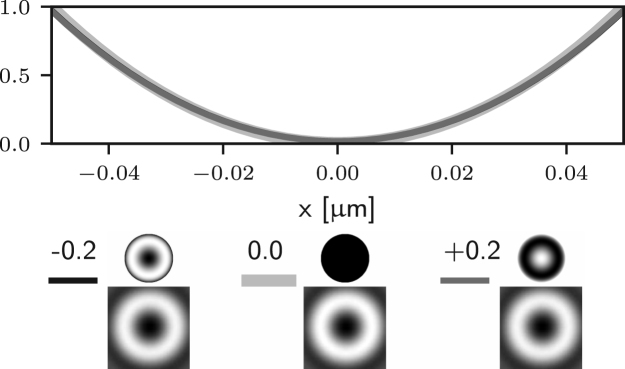
(a) $Z_{4}^{0}$.

**Fig. 5(b):**
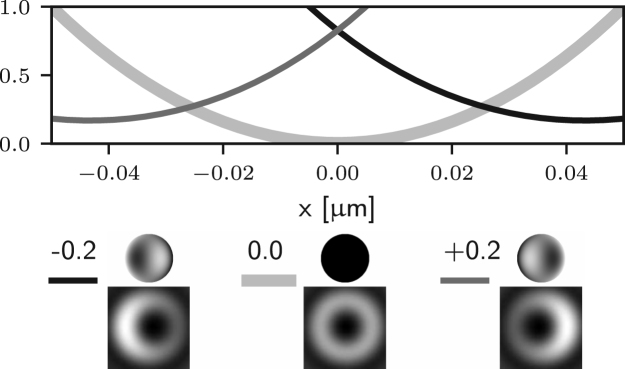
(b) $Z_{3}^{1}$.

**Fig. 5(c):**
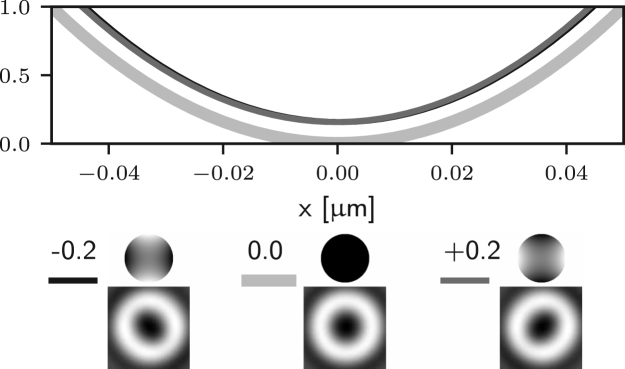
(c) $Z_{2}^{2}$.

**Fig. 5(d):**
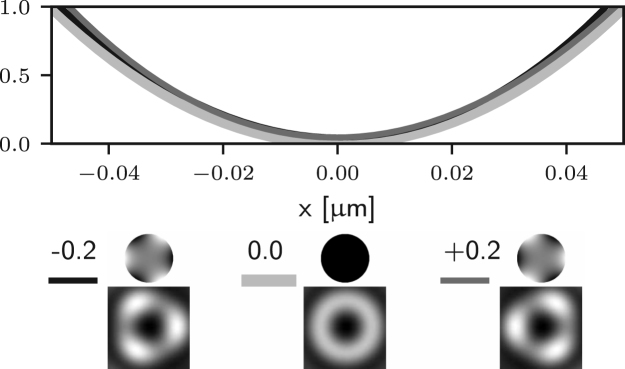
(d) $Z_{3}^{3}$.

## 7 Conclusion

The analysis has shown how, in the limit of small aberrations, the zero at the centre of the depletion focus is affected in different ways depending on the azimuthal order of the aberrations. These results are summarised in Table 1, where it can be seen that only certain modes can affect the intensity at the Gaussian focus of the depletion beam and that, in a subset of cases, the zero intensity is approximately maintained but shifted along the lateral plane or along the axis. It is important to emphasise that these conclusions apply at first order approximation, i.e., provided the magnitude of the aberrations is small enough so that the errors in approximating the GPF are negligible [27], [28]. Beyond this small aberration regime, the zero may also be affected when m≥4.

The information in Table 1 will be important for the design of adaptive optics schemes for STED microscopes. In particular, it is clear that in the small aberration regime, astigmatism plays a primary role in the suppression of signal from both 2D and 3D STED microscopes, whereas trefoil plays a lesser role in 3D STED. These modes should therefore be compensated before other modes. The approximate translation of the minimum intensity can also have a significant role on the performance of STED microscopes. Although we only model the effects of aberrations on the depletion focus in the present paper, it has been shown elsewhere [13] that small amounts of spherical aberration can shift the excitation and depletion foci apart, which results in a reduction of the fluorescence emission. A similar reduction in the emission is also found when considering small amounts of coma aberration in combined 2D/3D STED [24], caused by the two depletion foci moving in opposite directions. Although the discussion here is presented in the context of STED microscopy, we note that the results are equally relevant to similar techniques, such as RESOLFT microscopy [29] and super-resolution laser fabrication methods [30].
